# Safety of reduced antigen content diphtheria-tetanus-acellular pertussis vaccine when administered during pregnancy as part of the maternal immunization program in Brazil: a single center, observational, retrospective, cohort study

**DOI:** 10.1080/21645515.2019.1627161

**Published:** 2019-06-20

**Authors:** Mauro Sancovski, Narcisa Mesaros, Yang Feng, M. Angeles Ceregido, Dominique Luyts, Eliana De Barros

**Affiliations:** a Faculdade de Medicina do ABC, Santo André, Brazil; b GSK, Wavre, Belgium; c Ningyang Group Co., Limited, C/O GSK, Wavre, Belgium; d GSK, Rio de Janeiro, Brazil

**Keywords:** Pertussis, maternal vaccination, adverse event, pregnancy, neonate

## Abstract

Reduced antigen diphtheria-tetanus-acellular pertussis (Tdap) vaccination is included in the maternal immunization program in Brazil since September 2014. We investigated associations between maternal Tdap vaccination and pregnancy-related adverse events (AEs) (gestational diabetes, pregnancy-related hypertension, and pregnancy hemorrhage) and neonatal AEs of interest (preterm birth and small for gestational age). This descriptive, observational, retrospective, single-center study in Brazil (NCT02757950) compared data from medical charts of 1203 pregnant women who received Tdap as part of the maternal immunization program and delivered between May 2015 and February 2017 (exposed cohort) and 1259 unvaccinated women who delivered between September 2012 and August 2014 (unexposed cohort). Index dates were defined as the time of vaccination (27–39 gestational weeks; exposed cohort) or 27 gestational weeks (unexposed cohort). Cumulative incidences were calculated as the number of women with each event between index and delivery dates divided by the total number of women with vaccination date available in the exposed cohort (N = 1199) or the total number of women in the unexposed cohort (N = 1259). Cumulative incidences per 1000 persons were 8.34 versus 17.47 for gestational diabetes, 9.17 versus 24.62 for pregnancy-related hypertension, 3.34 versus 15.09 for pregnancy hemorrhage, 53.38 versus 96.11 for preterm birth, and 57.55 versus 49.25 for small for gestational age in the exposed versus unexposed cohorts. No increased risk of pregnancy-related AEs or neonatal AEs of interest was found following maternal vaccination with Tdap. These results should be interpreted cautiously due to limitations inherent to retrospective observational studies.

## Introduction

Pertussis is a highly contagious severe respiratory infection caused by *Bordetella pertussis*^1^. The risk of severe pertussis is higher in infants younger than 3 months of age due to their developing respiratory system and because they are too young to have completed their primary vaccination schedule against pertussis.^1^ Outbreaks occurred in the last decade, underlining the need for effective protection against pertussis through vaccination. For example, 9154 pertussis cases, of which 5482 were confirmed, were reported in the general population in California during an outbreak in 2010, and 9711 laboratory-confirmed cases occurred during an outbreak in 2012 in England and Wales.^1,2^

Pertussis disease patterns are cyclic with incidence peaks occurring every 3–4 y.^3^ Overall pertussis incidence has increased in many countries since 2010, including several high-income countries with high coverage of acellular pertussis vaccination in childhood.^1,2,4–8^ In Brazil, a systematic review of observational studies showed that pertussis disease incidence increased between 2010 and 2014, with an average incidence rate of 2.19 cases per 100 000 inhabitants and a peak of 4.03 cases per 100 000 inhabitants in 2014. A total of 415 deaths related to pertussis were recorded between 2010 and 2015 in Brazil, of which 97.6% occurred in children younger than 1 yof age.^9^

A 3-dose primary vaccination schedule with a whole-cell pertussis vaccine at 2, 4 and 6 months of age followed by a booster dose at 15 months of age was introduced in the National Immunization Program (NIP) in Brazil in 1977. A second booster dose at the age of 4–6 y was introduced in 2004.^3^ Due to the increases in pertussis incidence and fatal cases among infants, especially in those too young to be vaccinated, the NIP in Brazil implemented pertussis maternal immunization with 1 dose of combined reduced antigen content diphtheria-tetanus-acellular pertussis vaccine (Tdap) during the last trimester of pregnancy by the end of 2014.^10^ Besides protecting the mother, the objective of maternal immunization is to provide protection to the infants during the first months of life, mainly through the transfer of maternal antibodies to the infant by transplacental transport.^11–13^ Maternal transfer of immunoglobulin G begins as early as 13 weeks of gestation and rises continuously between 17 and 41 gestational weeks.^14–16^ Some countries, such as the UK, recommend maternal immunization from 16 weeks of gestation,^17^ while others, such as the United States (US), recommend to vaccinate pregnant women between 27 and 36 completed gestational weeks.^18^ To give access to vaccination to more women living in rural areas, the recommendation was changed in Brazil to start maternal immunization at 20 gestational weeks in 2017.^19^

**Figure 1.**
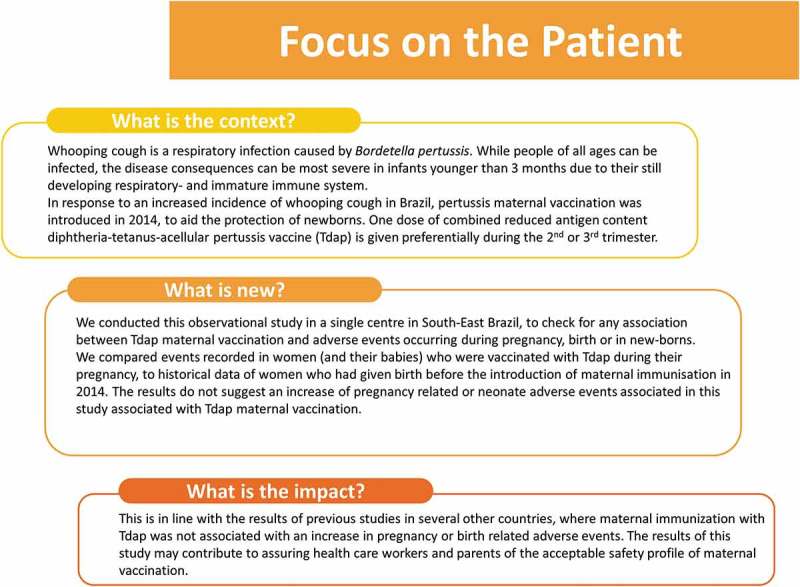
Focus on the patient.

In Brazil, a significant reduction in the number of suspected and confirmed pertussis cases was observed in 2015, following the inclusion of Tdap in the maternal immunization program and improvement in vaccine coverage.^9,20^ A decrease in the number of cases suggests that Tdap maternal immunization is an effective strategy to prevent pertussis disease in infants.^13,21,22^ Although a growing body of scientific evidence supports the safety of Tdap maternal immunization,^23,24^ the safety data currently available from South America are limited.^25^ In this context, the introduction of Tdap in the maternal immunization program in Brazil was a unique opportunity to investigate the association between routine maternal Tdap vaccination and pregnancy-related adverse events (AEs) and neonatal AEs of interest in a large cohort of pregnant women in this part of the world. Figure 1 summarizes the research, clinical relevance and impact of this study on the patient population.

## Results

### Characteristics of the study participants

**Figure 2.**
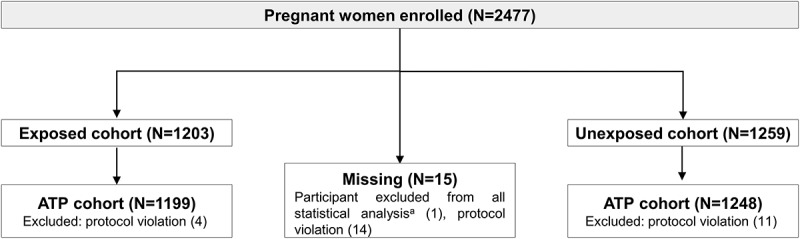
Flow of participants. **Notes**: Exposed cohort, pregnant women who had received Tdap as part of the maternal immunization program in Brazil; unexposed Cohort, women pregnant before implementation of the maternal immunization program in Brazil who did not receive Tdap. N, number of participants.Note: Participant may have more than one elimination code assigned.^a^Due to missing information (i.e., no data reported but subject number allocated).

A total of 2477 women were enrolled in the study (1203 in the exposed cohort, 1259 in the unexposed cohort, and 15 with missing information). Of those, 1199 women in the exposed cohort and 1248 women in the unexposed cohort were included in the according-to-protocol cohort (Figure 2). Since less than 5% of participants were eliminated from the total-enrolled cohort, no analyses were done on the according-to-protocol cohort.

Demographic characteristics were well-balanced between the exposed and unexposed cohorts in the total-enrolled cohort (Table 1). The mean age at the beginning of pregnancy was 26.38 y (standard deviation [SD]: 6.20 y).

**Table 1.** Demographic characteristics of study participants (total-enrolled cohort).

Characteristics	Exposed cohort *N* = 1203, *N*’ = 1199	Unexposed cohort *N* = 1259, *N*’ = 1248
**Resident of the study area, n (%)**	1199 (100)	1248 (100)
Age group at the beginning of pregnancy in years, ***n*** (%)		
18–19 y	147 (12.3)	198 (15.9)
20–24 y	395 (32.9)	365 (29.2)
25–29 y	301 (25.1)	311 (24.9)
30–34 y	201 (16.8)	222 (17.8)
35–39 y	124 (10.3)	120 (9.6)
≥40 y	31 (2.6)	32 (2.6)
**Age at the beginning of pregnancy in years, Mean (SD)**	26.49 (6.15)	26.28 (6.24)

**Abbreviations**: *N*, total number of participants; *N*’, number of participants with data available; *n* (%), number (percentage) of participants; SD, standard deviation.

### Co-primary objectives

In the total enrolled cohort, the cumulative incidences of gestational diabetes, pregnancy-related hypertension and pregnancy (vaginal) hemorrhage reported between index date and delivery date were 8.34, 9.17 and 3.34 cases per 1000 persons in the exposed cohort, and 17.47, 24.62 and 15.09 cases per 1000 persons in the unexposed cohort (Table 2). The cumulative incidences of preterm birth and small for gestational age were 53.38 and 57.55 cases per 1000 persons in the exposed cohort and 96.11 and 49.25 cases per 1000 persons in the unexposed cohort (Table 2).

**Table 2.** Cumulative incidence of pregnancy-related adverse events and neonatal AEs of interest in current pregnancy in the exposed and unexposed cohorts (total-enrolled cohort).

	Exposed cohort *N* = 1199		Unexposed cohort *N*= 1259	
Adverse event or birth outcome	*n*	Incidence proportion per 1000 (99% CI)	*n*	Incidence proportion per 1000 (99% CI)
Gestational diabetes	10	8.34 (3.10; 17.85)	22	17.47 (9.37; 29.56)
Pregnancy-related hypertension^a^	11	9.17 (3.60; 19.00)	31	24.62 (14.72; 38.47)
Pre-Eclampsia	10	8.34 (3.10; 17.85)	30	23.83 (14.11; 37.50)
Eclampsia	2	1.67 (0.09; 7.73)	0	0.00 (0.00; 4.21)
HELLP Syndrome	0	0.00 (0.00; 4.42)	1	0.79 (0.00; 5.90)
Vaginal hemorrhage	4	3.34 (0.56; 10.50)	19	15.09 (7.66; 26.52)
Preterm birth	64	53.38 (37.76; 73.09)	121	96.11 (75.10; 120.99)
Small for gestational age	69	57.55 (41.27; 77.92)	62	49.25 (34.63; 67.77)

**Abbreviations**: CI, confidence interval; *N*, number of participants at risk (unexposed cohort) and with vaccination date (exposed cohort); *n*, number of participants where at least one event that occurred (1) between index date and date of delivery for pregnancy-related adverse events and (2) after index date for birth outcome events (preterm birth and small for gestational age). Vaginal hemorrhage includes ante-partum, intra-partum, and post-partum hemorrhage.

Note: ^a^In the total cohort, 146 pregnant women (93 in the exposed cohort and 53 in the unexposed cohort) experienced gestational or pregnancy-related hypertension independently of pre-eclampsia or eclampsia recorded in the free text variable of the electronic case report form.

The unadjusted ORs between the exposed and unexposed cohorts and their 95% CIs were lower than 1 for gestational diabetes (0.47 [95% CI: 0.22–0.99]), pregnancy-related hypertension (0.37 [95% CI: 0.18–0.73]), pregnancy hemorrhage (0.22 [95% CI: 0.07–0.64]) and preterm birth (0.52 [95% CI: 0.38–0.71]). The unadjusted ORs between the exposed and unexposed cohorts were 1.16 (95% CI: 0.81–1.64) for small for gestational age (Supplementary Tables 1 and 2).

The results of the sensitivity analysis adjusted for follow-up period also showed lower incidence rates in the exposed than in the unexposed cohort for all primary endpoints, except for small for gestational age. The incidence rates of gestational diabetes, pregnancy-related hypertension and pregnancy (vaginal) hemorrhage reported between index date and delivery date were 0.93, 1.02 and 0.37 cases per 1000 person-weeks in the exposed cohort, and 1.69, 2.39 and 1.46 cases per 1000 person-weeks in the unexposed cohort. The incidence rates of preterm birth and small for gestational age were 5.94 and 6.41 cases per 1000 person-weeks in the exposed cohort and 9.32 and 4.78 cases per 1000 person-weeks in the unexposed cohort. The incidence rate ratios between exposed and unexposed cohort calculated using Poisson regression were 0.55 (95% CI: 0.26–1.16) for gestational diabetes, 0.43 (95% CI: 0.22–0.85) for pregnancy-related hypertension, 0.25 (95% CI: 0.09–0.75) for vaginal hemorrhage, 0.64 (95% CI: 0.47–0.86) for preterm birth and 1.34 (95% CI: 0.95–1.89) for small for gestational age.

The risk factors of pregnancy-related AEs and neonatal AEs of interest identified by the adjusted multiple logistic regression model included placenta abruption and gestational diabetes in previous pregnancy for gestational diabetes; pre-eclampsia in previous pregnancy and premature rupture of membranes in previous pregnancy for pregnancy-related hypertension; no maternal vaccination against Tdap during pregnancy, placenta previa and neonatal death in previous pregnancy for vaginal hemorrhage; no maternal vaccination against Tdap during pregnancy, a maternal age of 35 y and above compared with 20–24 y at the start of pregnancy and preterm baby in previous pregnancy for preterm birth; and smoking before or during pregnancy, premature rupture of membrane in previous pregnancy and neonatal hypoxic-ischemic encephalopathy in previous pregnancy for small baby for gestational age (Table 3). Of note, the inclusion of placenta previa and neonatal hypoxic-ischemic encephalopathy in previous pregnancy as risk factors for vaginal hemorrhage and for small baby for gestational age, respectively, should be interpreted with caution because very few events were reported, and 95% CIs were very large. The results of the univariate analysis performed to identify the possible risk factors are presented in Supplementary Table 1 for the pregnancy-related AEs and in Supplementary Table 2 for the neonatal AEs of interest.

**Table 3.** Estimated coefficients for the adjusted logistic regression model to explore the risk factors of pregnancy-related AEs and neonatal AEs of interest in current pregnancy (Total cohort).

Characteristic		Compared levels	Coefficient	Standard error	*P*-value	Adjusted OR (95% CI)
**Gestational diabetes**						
Independent factor		Continuous*	−4.2961	0.2442	<.0001	
Placenta abruption		Yes vs. No	2.3502	1.0966	0.0321	10.487 (1.223; 89.966)
Gestational diabetes in previous pregnancy		Yes vs. No	2.4243	0.7978	0.0024	11.294 (2.364; 53.948)
**Pregnancy-related hypertension**						
Independent factor		Continuous*	−4.3470	0.2516	<.0001	
Pre-eclampsia in previous pregnancy		Yes vs. No	1.7569	0.6493	0.0068	5.795 (1.623; 20.687)
Premature rupture of membranes in previous pregnancy		Yes vs. No	2.1498	1.0837	0.0473	8.583 (1.026; 71.793)
**Vaginal hemorrhage**						
Independent factor		Continuous*	−4.2397	0.3464	<.0001	
Tdap maternal vaccination during pregnancy		Exposed vs. Unexposed	−2.7016	1.1088	0.0148	0.067 (0.008; 0.590)
Placenta previa		Yes vs. No	5.5905	1.8417	0.0024	267.870 (7.249; >999.999)
Neonatal death in previous pregnancy		Yes vs. No	2.9178	0.8591	0.0007	18.501 (3.435; 99.646)
**Preterm birth**						
Independent factor		Continuous*	−3.2547	0.3566	<.0001	
Tdap maternal vaccination during pregnancy		Exposed vs. Unexposed	−0.5061	0.2756	0.0663	0.603 (0.351; 1.035)
Maternal age at the start of the pregnancy (in years)		18-19Y vs. 20-24Y	0.5188	0.6883	0.4510	1.680 (0.436; 6.474)
		25-29Y vs. 20-24Y	−0.1343	0.4530	0.7670	0.874 (0.360; 2.125)
		30-34Y vs. 20-24Y	0.6583	0.4114	0.1095	1.931 (0.862; 4.325)
		35-39Y vs. 20-24Y	1.0124	0.4300	0.0186	2.752 (1.185; 6.393)
		GE 40Y vs. 20-24Y	1.0044	0.7086	0.1564	2.730 (0.681; 10.949)
Pre-term baby (<37 weeks) in previous pregnancies		Yes vs. No	1.5389	0.2964	<.0001	4.659 (2.606; 8.330)
**Small for gestational age**						
Independent factor		Continuous*	−3.3069	0.1811	<.0001	
Smoking before and/or during pregnancy		Yes vs. No	0.8812	0.3773	0.0195	2.414 (1.152; 5.056)
Premature rupture of membranes in previous pregnancy		Yes vs. No	2.0789	0.8407	0.0134	7.996 (1.539; 41.539)
Neonatal hypoxic ischemic encephalopathy in previous pregnancy		Yes vs. No	3.3069	1.4258	0.0204	27.299 (1.669; 446.436)

**Abbreviation**s: AE, adverse event; OR, odds ratio; 95% CI, 95% confidence interval; Y, years. *OR for continuous variables describes the effect of a difference of one unit.

### Secondary objectives

#### Other pregnancy-related AEs and neonatal AEs of interest

The cumulative incidences of premature rupture of membranes, preterm premature rupture of membranes, premature uterine contraction and stillbirth were 158.47, 14.18, 26.69 and 0.83 cases per 1000 persons in the exposed cohort, and 207.31, 28.59, 42.89 and 4.77 cases per 1000 persons in the unexposed cohort (Table 4). While the cumulative incidence of neonatal death was 6.35 cases per 1000 persons in the unexposed cohort, there were no cases in the exposed cohort. There were no events of maternal death and neonatal hypoxic-ischemic encephalopathy reported in both the exposed and the unexposed cohort.

**Table 4.** Cumulative incidence of other pregnancy-related adverse events and neonatal AEs of interest in current pregnancy in the exposed and unexposed cohorts (total-enrolled cohort).

	Exposed cohort *N* = 1199		Unexposed cohort *N* = 1259	
Adverse event or birth outcome	*n*	Incidence proportion per 1000 (95% CI) (95% CI)	*n*	Incidence proportion per 1000 (95% CI)
Premature rupture of membranes	190	158.47 (136.73; 182.67)	261	207.31 (182.92; 234.04)
Preterm premature rupture of membranes	17	14.18 (8.26; 22.70)	36	28.59 (20.03; 39.59)
Premature uterine contraction	32	26.69 (18.26; 37.68)	54	42.89 (32.22; 55.96)
Neonatal death	0	0.00 (0.00; 3.08)	8	6.35 (2.74; 12.52)
Maternal death	0	0.00 (0.00; 3.08)	0	0.00 (0.00; 2.93)
Stillbirth	1	0.83 (0.02; 4.65)	6	4.77 (1.75; 10.37)
Neonatal hypoxic ischemic encephalopathy	0	0.00 (0.00; 3.08)	0	0.00 (0.00; 2.93)
Congenital anomalies	5	4.17 (1.35; 9.73)	22	17.47 (10.95; 26.46)

**Abbreviation**s: CI, confidence interval; *N*, number of participants at risk (unexposed cohort) and with vaccination date in the exposed cohort; *n*, number of participants where at least one event occurred (1) between index date and date of delivery for pregnancy-related adverse events and (2) after index date for neonate-related events (neonatal death, stillbirth, neonatal hypoxic-ischemic encephalopathy, and congenital anomalies).

#### Congenital anomalies

The cumulative incidence of congenital anomalies was 4.17 cases per 1000 persons (95% CI: 1.35–9.73) in the exposed cohort and 17.47 cases per 1000 persons (95% CI: 10.95–26.46) in the unexposed cohort (Table 4).

Congenital anomalies were reported in 22 neonates born to women from the unexposed cohort and in 5 neonates born to women from the exposed cohort (Supplementary Table 3). None of the congenital anomalies reported in the exposed cohort was considered related to the administration of Tdap to the mother during pregnancy.

#### Pregnancy-related AEs and neonatal AEs of interest per study year

In the unexposed cohort, the cumulative incidences of most pregnancy-related AEs and neonatal AEs of interest recorded between September 2012 and August 2013 were comparable to those recorded between September 2013 and August 2014 (Supplementary Table 4).

However, the cumulative incidences of pregnancy hemorrhage and preterm birth decreased from 26.02 (95% CI: 14.87–42.25) and 107.32 (95% CI: 83.00–136.53) cases per 1000 persons between September 2012 and August 2013 to 4.74 (95% CI: 0.98–13.85) and 86.89 (95% CI: 65.46–113.10) per 1000 persons between September 2013 and August 2014, respectively.

## Discussion

We present the results of a retrospective study, which included 1203 vaccinated and 1259 unvaccinated pregnant women and was conducted in the immediate period after the introduction of Tdap in the maternal immunization program in Brazil. We found no association between vaccination with Tdap during the third trimester of pregnancy and the specific pregnancy-related AEs or neonatal AEs of interest evaluated in this study.

In women who received maternal vaccination with Tdap compared to the unvaccinated historical cohort in Brazil, we identified no increased risk for the pregnancy-related AEs included in the primary objectives of this study (gestational diabetes, pregnancy-related hypertension and pregnancy hemorrhage) and for other pregnancy-related AEs (premature rupture of membranes, preterm premature rupture of membranes, premature uterine contraction and maternal death). These findings are in line with a growing body of scientific evidence on the safety of Tdap vaccination during pregnancy.^23,24,26–40^ The absence of increased risk for gestational diabetes is in line with the results of a previous retrospective-matched cohort study conducted in 53 885 Tdap exposed pregnant and 109 253 unexposed women in the US.^37^ Additionally, two systematic reviews,^33,35^ a retrospective cohort study including 26 229 vaccinated women in California,^28^ and a large observational, retrospective cohort study conducted in the UK in 20 074 pregnant women^36^ also found that the risk for hypertensive disorders did not increase in women who received antenatal pertussis vaccination. In the previous cohort study conducted in the UK, no increased risk for hemorrhage, maternal death, pre-eclampsia, eclampsia, and uterine rupture was observed following Tdap vaccination, which further corroborates our findings.^36^ Finally, the absence of increased risk for premature rupture of membranes, preterm premature rupture of membranes, and premature uterine contraction was consistent with findings of the systematic review of McMillan *et al*., showing no statistically or clinically significant risk for preterm labor following antenatal immunization with any antigen present in combination pertussis vaccines.^33^

Furthermore, our study found no increased risk for the neonatal AEs of interest included in the primary objectives (preterm birth and small for gestational age) and other neonatal AEs (neonatal death, stillbirth, neonatal hypoxic-ischemic encephalopathy, and congenital anomalies) following the introduction of Tdap in the maternal immunization program in Brazil. The incidence of small for gestational age was slightly higher in the neonates born to mother who received Tdap vaccination compared to the unvaccinated cohort, but the difference was not statistically significant and could be affected by the variability of the information encoded in the medical records. Therefore, these results should be interpreted with caution. Moreover, no increased risk for small for gestational age was observed in neonates born to vaccinated women in a previous review by McMillan *et al.*,^33^ and a study based on large US insurance claims databases.^28^ The absence of increased risk of preterm birth after antenatal pertussis exposure observed in our study was in line with findings of previous systematic reviews,^33,35^ a study based on large US insurance claims databases^28^ and prospective observational studies conducted in New-Zealand.^30,31^ A retrospective study in the US showed significant increases in preterm birth rates in infants born to women who declined (226 women) Tdap vaccination compared to those who accepted (7152 women).^29^ The absence of increased risk for neonatal death and/or stillbirth in exposed compared with unexposed women was also shown in the systematic review by Futura *et al*.^35^ and observational studies in the UK,^36^ the US^29^ and New-Zealand.^30,31^ Finally, our results corroborated also those of the systematic review by McMillan *et al.*,^33^ retrospective studies in the US^26,29,41^ and prospective observational studies conducted in New-Zealand^30,31^ suggesting that Tdap vaccination during pregnancy did not increase the risk of congenital anomalies. In our study, the incidence of congenital anomalies was lower in infants born to women who received Tdap vaccination during pregnancy compared to infants born to unexposed women. Potential explanations for this observation include potential confounding by women with high-risk pregnancies being potentially less likely to be vaccinated; however, the reasons for not vaccinating were out of the scope of this study. In our study, two cases of microcephaly were identified in infants born to women who received Tdap during pregnancy, which were not considered to be causally related to vaccination. A previous study has shown that maternal Tdap vaccination was not significantly associated with increased risk for microcephaly.^41^ Although the substantial increase in the number of microcephaly cases observed in 2015 in Brazil was probably due to increases in maternal Zika virus infections,^41,42^ the two children with microcephaly in our study were born to mothers who were not infected by this virus during pregnancy.

This retrospective study presented a unique opportunity to compare the safety of Tdap in a large cohort of vaccinated pregnant women, in the period following its introduction in the maternal immunization program, with a historical cohort of unvaccinated pregnant women. Although our results are in line with the current knowledge on the safety profile of Tdap vaccines during pregnancy, they should be interpreted with caution in the light of study limitations. This study was limited by the average longer follow-up period in the unexposed cohort, having a gestational age of 27 weeks as index date, compared with the exposed cohort, having the vaccination date as index date; a sensitivity analysis showed that the difference in follow-up duration had a limited impact on the conclusions. Additional potential biases included the inaccurate capture of the timing of events with respect to Tdap vaccination, the incompleteness and imprecision of medical records, the influence of maternal Tdap vaccination program on the attitude of pregnant women towards attending antenatal care or medical consultation and on the frequency of pregnancy-related AEs reporting. Other drawbacks were the unavailability of information on concomitant vaccination administered during pregnancy for the unexposed cohort, and the variability between physicians to identify, classify and report adverse pregnancy outcomes when data are retrieved from secondary sources. This variability of the information encoded in the medical records may also affect the lower incidence of some AEs in vaccinated versus unvaccinated women, which cannot be directly attributed to the effect of the vaccine. Since it is estimated that 30% of congenital anomalies are diagnosed up to 6 months of age,^43^ an additional limitation of our study was the fact that congenital anomalies were only collected when the diagnosis was done at birth. Another drawback was the potential bias introduced by changes over time and confounding factors, which was the reason for performing analyses per study year in the historical-unvaccinated cohort. These analyses showed a decrease in the incidence of pregnancy hemorrhage and preterm birth, but these results should be interpreted with caution since there was no difference in standard of care provided to pregnant women and no explanation for this observation has been identified. A further limitation was the fact that the cohort of exposed women could not be compared with a contemporary parallel cohort of unvaccinated pregnant women due to the expected high vaccination coverage rate at the time of the study.

Finally, the exposed cohort included women vaccinated between 27 and 36 weeks of gestation while the recommendation in Brazil changed in 2017 to 20 weeks onwards. This may limit the extrapolation of the study results in Brazil, but is in line with the gestational window for vaccination recommended in several countries worldwide.

In summary, we found no increased risk of specific pregnancy-related AEs and neonatal AEs of interest in a cohort of women who received Tdap vaccination during pregnancy following its introduction in the maternal immunization program in Brazil compared with a historical cohort of unvaccinated pregnant women. These results should be interpreted cautiously in light of study limitations inherent to this retrospective observational study.

## Methods

### Study design and population

This post-authorization, observational, retrospective, cohort, safety study was conducted in one center in São Bernardo do Campo, São Paulo, Brazil. The study population consisted of 2 cohorts: one including pregnant women who received Tdap (*Refortrix*, the brand name of *Boostrix* in Brazil, GSK) as part of the maternal immunization program in Brazil and delivered between May 2015 and February 2017 (exposed cohort), and a historical cohort of unvaccinated pregnant women who delivered between September 2012 and August 2014, before the implementation of the maternal immunization program (unexposed cohort). Pregnancy-related data were collected retrospectively between July 2016 and May 2017. A different selection process was used for the unexposed and the exposed cohorts to control for potential changes in standard of care over time. For the unexposed cohort, the total cohort had to be equally divided over the 2 y. Therefore, women were randomly selected from the list of pregnant women admitted for delivery each month for 2 y. To reach the required number of 600 women per year, the random selection continued until 50 eligible women were identified in a given month. For the exposed cohort, all women who delivered from May 2015 onwards at the center were considered sequentially for enrolment until the total number of 1200 was reached.

Study participants were women between 18 and 45 y of age at the time of pregnancy, who delivered in the study center, were residents of the city of São Bernardo do Campo, were compliant with the routine antenatal care, and had complete and relevant medical records available. Women in the exposed cohort received 1 dose of Tdap between 27 and 36 completed weeks of pregnancy (or as late as 20 d before delivery due date) as part of the maternal immunization program in Brazil and had appropriate vaccination records. Women in the unexposed cohort did not receive Tdap vaccination during pregnancy to the best knowledge of the investigator. Women were excluded from the study if they had been transferred to other specialized centers where their medical records would be inaccessible for the study.

The study protocol and other information requiring pre-approval were reviewed and approved by a national Independent Ethics Committee (IEC). This study was conducted in accordance with “good pharmacovigilance practices/good clinical practices” guidelines and all applicable regulatory requirements, including the Declaration of Helsinki. The study is registered at www.clinicaltrials.gov (NCT02757950) and a protocol summary is available at https://www.gsk-clinicalstudyregister.com/study (Study ID: 203153).

### Study objectives

The co-primary objectives were to compare the risk of gestational diabetes, pregnancy-related hypertension (including pre-eclampsia, eclampsia and HELLP syndrome), and pregnancy hemorrhage (ante-partum, intra-partum or post-partum) in women from the exposed and unexposed cohorts, and the risk of preterm birth and small for gestational age in neonates born to women from the exposed and unexposed cohorts.

The secondary objectives were to describe, in the exposed and unexposed cohorts, the risk of other pregnancy-related AEs/neonatal AEs of interest (premature rupture of membranes, preterm premature rupture of membranes, premature uterine contraction, neonatal death, maternal death, stillbirth and neonatal hypoxic-ischemic encephalopathy) and the risk of congenital anomalies in neonates, and to describe, in the unexposed cohort, the risk of pregnancy-related AEs and neonatal AEs of interest per calendar year.

### Data collection and analysis

Data collection was done via electronic case report forms based on individual medical chart reviews. Medical files and other hospital documents were used for collecting demographic data, medical/gynecological history, pregnancy-related AEs and neonatal AEs of interest. Pregnancy-related AEs and neonatal AEs of interest were diagnosed by investigators based on validated guidelines developed and published by a panel of experts.^44^ Since all hemorrhage data from the electronic case report forms were collected together in the final database, it was not possible to categorize them and to perform separate analyses for ante-partum, intra-partum, and post-partum hemorrhage.

The follow-up period started at 27 weeks of gestation for women in the unexposed cohort and at the time of vaccination (27–39 weeks of gestation) for women in the exposed cohort (index date) and ended at the delivery date. Vaccination occurred between 27 and 36 weeks of pregnancy, except for 2 women who received the Tdap vaccine at 37 weeks and 39 weeks of gestation, respectively.

The age of the mother was considered as an important risk factor for the occurrence of the pregnancy-related AEs of interest and was controlled in the risk factor analyses. The other risk factors potentially associated with onset of the outcomes of interest included parity, infection during current pregnancy, placenta previa, placenta abruption, alcohol consumption, substance abuse and smoking before and during pregnancy, and congenital anomalies in parents and first-degree relatives. The other risk factors potentially associated with onset of the outcomes of interest were related to previous pregnancies and included history of pregnancy-related hypertension, pre-eclampsia, eclampsia, HELLP syndrome, infection, gestational diabetes, vaginal hemorrhage, premature rupture of membranes, preterm premature rupture of membranes, premature uterine contraction, neonatal death, neonatal hypoxic-ischemic encephalopathy, newborn with low birth weight, fetal macrosomia and preterm delivery.

### Statistical analysis

Using a 2-sided (alpha = 0.01) test, and assuming a ratio of participants in the exposed to the unexposed cohort of 1:1 and a background proportion of events of 3% for both co-primary and secondary endpoints in the unexposed cohort, 2400 participants were needed to have more than 80% power to detect a relative risk of 2 or higher.

The main analysis for co-primary objectives was performed on women from the exposed cohort with a full vaccination date available and women from the unexposed cohort. The cumulative incidence for each specific primary endpoint was calculated with its exact 99% confidence interval (CI) as the number of women who had the event between the index date and the delivery date divided by the total number of women at risk for both the exposed and unexposed cohorts. The incidence rate ratio between the exposed and unexposed cohorts was obtained for each endpoint with its 2-sided 95% CI by means of a Poisson regression model using the Tdap vaccination status during this pregnancy as a binary independent variable. Absence of increased relative risk was concluded if the respective 95% CI contained 1.

A univariate analysis was performed for each primary safety event reported after the index date to identify the possible risk factors (unadjusted odds ratios [ORs]). Then, a multiple logistic regression model was fitted with the identified possible risk factors using an alpha level of 0.1. Adjusted ORs and their 95% CIs were derived from the final model. Missing or non-evaluable primary and secondary outcome measurements were not replaced, and women with missing or non-evaluable data were excluded from the main analysis.

Due to the different lengths of follow-up between the exposed and unexposed cohorts, incidence rates (number of participants per 1000 person-weeks) were computed for the co-primary endpoints as a planned sensitivity analysis, with number of participants referring to women with at least one event occurring between index date and delivery date.

## Figures and Tables

**Table:** 

AE	adverse event
CI	confidence interval
IEC	Independent Ethics Committee
OR	odds ratio
SD	standard deviation
Tdap	combined diphtheria-tetanus-acellular pertussis vaccine
UK	United Kingdom
US	United States

## Trademark statement

*Refortrix* and *Boostrix* trademarks are owned by the GSK group of companies. *Refortrix* is a trademark specific to Brazil.
