# Supplementary material for: Safety of reduced antigen content diphtheria-tetanus-acellular pertussis vaccine when administered during pregnancy as part of the maternal immunization program in Brazil: a single center, observational, retrospective, cohort study
Source: Hum Vaccin Immunother. 2019 Jun 20;15(12):2873–81. doi: 10.1080/21645515.2019.1627161 (PMC6930109; doi:10.1080/21645515.2019.1627161)
Supplement: Supplemental Material [file khvi-15-12-1627161-s001.zip › Supplementary table 1.docx]

**Supplementary table 1.** Estimated unadjusted odds ratio for exploring the risk factors of pregnancy-related adverse events in current pregnancy (Total cohort)

|  | | |  | **Gestational diabetes** | | |  | **Pregnancy-related hypertension** | | |  | **Vaginal hemorrhage** | | |
| --- | --- | --- | --- | --- | --- | --- | --- | --- | --- | --- | --- | --- | --- | --- |
| **Characteristic** |  | **Compared levels** |  | **Unadjusted OR (95% CI)** |  | **p-value** |  | **Unadjusted OR (95% CI)** |  | **p-value** |  | **Unadjusted OR (95% CI)** |  | **p-value** |
| Exposure status |  | Exposed vs. Unexposed |  | 0.466 (0.220; 0.988) |  | 0.0464 |  | 0.365 (0.183; 0.730) |  | 0.0043 |  | 0.217 (0.073; 0.638) |  | 0.0055 |
| Maternal age at the start of the pregnancy (in years) |  | 18-19Y vs. 20-24Y |  | 1.093 (0.199; 5.998) |  | 0.9182 |  | 0.876 (0.273; 2.814) |  | 0.8244 |  | 1.771 (0.473; 6.637) |  | 0.3963 |
|  |  | 25-29Y vs. 20-24Y |  | 1.560 (0.417; 5.834) |  | 0.5089 |  | 1.376 (0.581; 3.263) |  | 0.4682 |  | 1.495 (0.454; 4.922) |  | 0.5083 |
|  |  | 30-34Y vs. 20-24Y |  | 5.131 (1.623; 16.216) |  | 0.0054 |  | 1.454 (0.569; 3.713) |  | 0.4338 |  | 1.079 (0.256; 4.536) |  | 0.9178 |
|  |  | 35-39Y vs. 20-24Y |  | 6.697 (1.998; 22.445) |  | 0.0021 |  | 2.923 (1.174; 7.281) |  | 0.0212 |  | 2.517 (0.671; 9.448) |  | 0.1714 |
|  |  | GE 40Y vs. 20-24Y |  | 6.819 (1.222; 38.052) |  | 0.0286 |  | <0.001 (<0.001; >999.999) |  | 0.9842 |  | 2.436 (0.280; 21.173) |  | 0.4198 |
| Parity |  | Multiparous vs. Nulliparous |  | 2.225 (0.296; 16.710) |  | 0.4368 |  | 0.711 (0.208; 2.434) |  | 0.5872 |  | >999.999 (<0.001; >999.999) |  | 0.9737 |
| Parity (Nulliparous + Multiparous) |  | 1 vs. 0 |  | 2.310 (0.298; 17.917) |  | 0.4230 |  | 0.453 (0.116; 1.773) |  | 0.2552 |  | >999.999 (<0.001; >999.999) |  | 0.9586 |
|  |  | 2 vs. 0 |  | 1.531 (0.170; 13.826) |  | 0.7042 |  | 1.035 (0.271; 3.958) |  | 0.9600 |  | >999.999 (<0.001; >999.999) |  | 0.9647 |
|  |  | 3-4 vs. 0 |  | 2.851 (0.293; 27.749) |  | 0.3668 |  | 0.951 (0.189; 4.795) |  | 0.9518 |  | 1.000 (<0.001; >999.999) |  | 1.0000 |
|  |  | ≥5 vs. 0 |  | 7.882 (0.471; 131.893) |  | 0.1509 |  | 2.686 (0.264; 27.297) |  | 0.4035 |  | 1.000 (<0.001; >999.999) |  | 1.0000 |
| Infection during current pregnancy |  | Yes vs. NO |  | 0.645 (0.264; 1.574) |  | 0.3351 |  | 0.763 (0.363; 1.603) |  | 0.4749 |  | 0.783 (0.289; 2.117) |  | 0.6293 |
| Placenta previa |  | Yes vs. NO |  | <0.001 (<0.001; >999.999) |  | 0.9891 |  | <0.001 (<0.001; >999.999) |  | 0.9876 |  | 18.318 (2.116; 158.557) |  | 0.0083 |
| Placenta abruption |  | Yes vs. NO |  | 6.933 (0.868; 55.356) |  | 0.0678 |  | 5.274 (0.666; 41.783) |  | 0.1154 |  | 9.972 (1.234; 80.593) |  | 0.0310 |
| Alcohol consumption before and/or during pregnancy |  | Yes vs. NO |  | 1.799 (0.240; 13.497) |  | 0.5677 |  | 2.713 (0.635; 11.586) |  | 0.1779 |  | <0.001 (<0.001; >999.999) |  | 0.9845 |
| Substance abuse before and/or during pregnancy |  | Yes vs. NO |  | <0.001 (<0.001; >999.999) |  | 0.9869 |  | <0.001 (<0.001; >999.999) |  | 0.9902 |  | <0.001 (<0.001; >999.999) |  | 0.9890 |
| Smoking before and/or during pregnancy |  | Yes vs. NO |  | 0.621 (0.147; 2.619) |  | 0.5164 |  | 0.693 (0.213; 2.261) |  | 0.5435 |  | 0.411 (0.055; 3.061) |  | 0.3854 |
| Pregnancy-related hypertension in previous pregnancy |  | Yes vs. NO |  | 1.256 (0.165; 9.566) |  | 0.8256 |  | 4.753 (1.345; 16.794) |  | 0.0155 |  | 2.144 (0.272; 16.915) |  | 0.4692 |
| Pre-eclampsia in previous pregnancy |  | Yes vs. NO |  | 1.430 (0.187; 10.921) |  | 0.7300 |  | 5.495 (1.548; 19.506) |  | 0.0084 |  | 2.429 (0.307; 19.209) |  | 0.4001 |
| Eclampsia in previous pregnancy |  | Yes vs. NO |  | <0.001 (<0.001; >999.999) |  | 0.9928 |  | <0.001 (<0.001; >999.999) |  | 0.9928 |  | <0.001 (<0.001; >999.999) |  | 0.9945 |
| HELLP in previous pregnancy |  | Not performed |  | . |  | . |  | . |  | . |  | - |  | - |
| Infection in previous pregnancy |  | Yes vs. NO |  | <0.001 (<0.001; >999.999) |  | 0.9902 |  | <0.001 (<0.001; >999.999) |  | 0.9903 |  | <0.001 (<0.001; >999.999) |  | 0.9925 |
| Gestational diabetes in previous pregnancy |  | Yes vs. NO |  | 10.727 (2.255; 51.031) |  | 0.0029 |  | <0.001 (<0.001; >999.999) |  | 0.9872 |  | <0.001 (<0.001; >999.999) |  | 0.9896 |
| Vaginal hemorrhage in previous pregnancy |  | Yes vs. NO |  | <0.001 (<0.001; >999.999) |  | 0.9898 |  | <0.001 (<0.001; >999.999) |  | 0.9898 |  | <0.001 (<0.001; >999.999) |  | 0.9922 |
| Premature rupture of membranes in previous pregnancy |  | Yes vs. NO |  | <0.001 (<0.001; >999.999) |  | 0.9907 |  | 7.462 (0.900; 61.861) |  | 0.0625 |  | <0.001 (<0.001; >999.999) |  | 0.9928 |
| Preterm premature rupture of membranes in previous pregnancy |  | Yes vs. NO |  | <0.001 (<0.001; >999.999) |  | 0.9858 |  | <0.001 (<0.001; >999.999) |  | 0.9859 |  | 5.293 (0.655; 42.799) |  | 0.1181 |
| Premature uterine contraction in previous pregnancy |  | Yes vs. NO |  | 2.887 (0.651; 12.803) |  | 0.1631 |  | 1.358 (0.178; 10.343) |  | 0.7679 |  | 2.327 (0.294; 18.381) |  | 0.4233 |
| Neonatal death in previous pregnancy |  | Yes vs. NO |  | <0.001 (<0.001; >999.999) |  | 0.9890 |  | <0.001 (<0.001; >999.999) |  | 0.9886 |  | 19.846 (3.953; 99.634) |  | 0.0003 |
| Neonatal hypoxic ischemic encephalopathy in previous pregnancy |  | Yes vs. NO |  | <0.001 (<0.001; >999.999) |  | 0.9937 |  | <0.001 (<0.001; >999.999) |  | 0.9937 |  | <0.001 (<0.001; >999.999) |  | 0.9952 |
| New born with low birth weight (<2.5 kg) in previous pregnancy |  | Yes vs. NO |  | 1.825 (0.508; 6.556) |  | 0.3567 |  | 2.294 (0.736; 7.146) |  | 0.1521 |  | 0.652 (0.083; 5.091) |  | 0.6832 |
| Fetal macrosomia (newborn > 4 kg) in previous pregnancies |  | Yes vs. NO |  | 1.646 (0.212; 12.808) |  | 0.6339 |  | <0.001 (<0.001; >999.999) |  | 0.9798 |  | <0.001 (<0.001; >999.999) |  | 0.9831 |
| Pre-term baby (<37 weeks) in previous pregnancies |  | Yes vs. NO |  | 2.028 (0.652; 6.305) |  | 0.2220 |  | 1.302 (0.372; 4.552) |  | 0.6796 |  | 0.719 (0.091; 5.716) |  | 0.7554 |

Footnote: OR, odds ratio; 95% CI, 95% confidence interval; Y, years. Vaginal hemorrhage includes ante-partum, intra-partum and post-partum hemorrhage during the whole pregnancy.
