# Supplementary material for: Safety of reduced antigen content diphtheria-tetanus-acellular pertussis vaccine when administered during pregnancy as part of the maternal immunization program in Brazil: a single center, observational, retrospective, cohort study
Source: Hum Vaccin Immunother. 2019 Jun 20;15(12):2873–81. doi: 10.1080/21645515.2019.1627161 (PMC6930109; doi:10.1080/21645515.2019.1627161)
Supplement: Supplemental Material [file khvi-15-12-1627161-s001.zip › Supplementary table 2.docx]

**Supplementary table 2.** Estimated unadjusted odds ratio for exploring the risk factors for neonatal adverse events of interest in current pregnancy (Total cohort)

|  | | |  | **Preterm birth** | | |  | **Small for gestational age** | | |
| --- | --- | --- | --- | --- | --- | --- | --- | --- | --- | --- |
| **Characteristic** |  | **Compared levels** |  | **Unadjusted OR (95% CI)** |  | **p-value** |  | **Unadjusted OR (95% CI)** |  | **p-value** |
| Exposure status |  | Exposed vs. Unexposed |  | 0.520 (0.380; 0.712) |  | <.0001 |  | 1.155 (0.812; 1.644) |  | 0.4224 |
| Maternal age at the start of the pregnancy (in years) |  | 18-19Y vs. 20-24Y |  | 1.325 (0.809; 2.170) |  | 0.2638 |  | 1.266 (0.738; 2.170) |  | 0.3912 |
|  |  | 25-29Y vs. 20-24Y |  | 1.261 (0.826; 1.925) |  | 0.2836 |  | 1.155 (0.724; 1.840) |  | 0.5454 |
|  |  | 30-34Y vs. 20-24Y |  | 1.438 (0.914; 2.263) |  | 0.1165 |  | 0.823 (0.464; 1.457) |  | 0.5029 |
|  |  | 35-39Y vs. 20-24Y |  | 1.709 (1.020; 2.865) |  | 0.0418 |  | 1.050 (0.551; 2.001) |  | 0.8828 |
|  |  | GE 40Y vs. 20-24Y |  | 1.627 (0.666; 3.972) |  | 0.2851 |  | 0.921 (0.276; 3.067) |  | 0.8927 |
| Parity |  | Multiparous vs. Nulliparous |  | 0.352 (0.207; 0.600) |  | 0.0001 |  | 0.715 (0.332; 1.538) |  | 0.3905 |
| Parity (Nulliparous + Multiparous) |  | 1 vs. 0 |  | 0.422 (0.242; 0.738) |  | 0.0025 |  | 0.869 (0.395; 1.913) |  | 0.7281 |
|  |  | 2 vs. 0 |  | 0.260 (0.129; 0.524) |  | 0.0002 |  | 0.419 (0.158; 1.108) |  | 0.0795 |
|  |  | 3-4 vs. 0 |  | 0.250 (0.097; 0.643) |  | 0.0040 |  | 0.802 (0.283; 2.272) |  | 0.6773 |
|  |  | ≥5 vs. 0 |  | 0.353 (0.044; 2.801) |  | 0.3245 |  | <0.001 (<0.001; >999.999) |  | 0.9860 |
| Infection during current pregnancy |  | Yes vs. NO |  | 0.785 (0.548; 1.125) |  | 0.1874 |  | 0.582 (0.368; 0.921) |  | 0.0209 |
| Placenta previa |  | Yes vs. NO |  | <0.001 (<0.001; >999.999) |  | 0.9829 |  | <0.001 (<0.001; >999.999) |  | 0.9856 |
| Placenta abruption |  | Yes vs. NO |  | 5.284 (1.355; 20.607) |  | 0.0165 |  | 1.961 (0.247; 15.593) |  | 0.5244 |
| Alcohol consumption before and/or during pregnancy |  | Yes vs. NO |  | 1.528 (0.596; 3.919) |  | 0.3779 |  | 1.729 (0.610; 4.903) |  | 0.3030 |
| Substance abuse before and/or during pregnancy |  | Yes vs. NO |  | 2.598 (0.875; 7.717) |  | 0.0855 |  | 2.675 (0.785; 9.121) |  | 0.1158 |
| Smoking before and/or during pregnancy |  | Yes vs. NO |  | 0.913 (0.544; 1.533) |  | 0.7317 |  | 2.032 (1.269; 3.255) |  | 0.0032 |
| Pregnancy-related hypertension in previous pregnancy |  | Yes vs. NO |  | 1.336 (0.469; 3.802) |  | 0.5873 |  | 0.824 (0.196; 3.467) |  | 0.7913 |
| Pre-eclampsia in previous pregnancy |  | Yes vs. NO |  | 1.525 (0.533; 4.363) |  | 0.4314 |  | 0.936 (0.221; 3.952) |  | 0.9278 |
| Eclampsia in previous pregnancy |  | Yes vs. NO |  | <0.001 (<0.001; >999.999) |  | 0.9862 |  | <0.001 (<0.001; >999.999) |  | 0.9878 |
| HELLP in previous pregnancy |  | Not performed |  |  |  |  |  |  |  |  |
| Infection in previous pregnancy |  | Yes vs. NO |  | 3.729 (0.791; 17.574) |  | 0.0961 |  | <0.001 (<0.001; >999.999) |  | 0.9892 |
| Gestational diabetes in previous pregnancy |  | Yes vs. NO |  | 2.817 (0.811; 9.785) |  | 0.1030 |  | 3.737 (1.069; 13.064) |  | 0.0390 |
| Vaginal hemorrhage in previous pregnancy |  | Yes vs. NO |  | 1.504 (0.192; 11.804) |  | 0.6980 |  | <0.001 (<0.001; >999.999) |  | 0.9887 |
| Premature rupture of membranes in previous pregnancy |  | Yes vs. NO |  | <0.001 (<0.001; >999.999) |  | 0.9883 |  | 5.549 (1.152; 26.738) |  | 0.0327 |
| Preterm premature rupture of membranes in previous pregnancy |  | Yes vs. NO |  | 6.230 (2.381; 16.300) |  | 0.0002 |  | 2.093 (0.479; 9.144) |  | 0.3264 |
| Premature uterine contraction in previous pregnancy |  | Yes vs. NO |  | 3.075 (1.329; 7.119) |  | 0.0087 |  | 0.460 (0.062; 3.392) |  | 0.4459 |
| Neonatal death in previous pregnancy |  | Yes vs. NO |  | 1.271 (0.164; 9.842) |  | 0.8186 |  | <0.001 (<0.001; >999.999) |  | 0.9878 |
| Neonatal hypoxic ischemic encephalopathy in previous pregnancy |  | Yes vs. NO |  | <0.001 (<0.001; >999.999) |  | 0.9880 |  | 21.951 (1.356; 355.415) |  | 0.0297 |
| New born with low birth weight (<2.5 kg) in previous pregnancy |  | Yes vs. NO |  | 2.339 (1.248; 4.382) |  | 0.0080 |  | 1.543 (0.704; 3.380) |  | 0.2786 |
| Fetal macrosomia (newborn > 4 kg) in previous pregnancies |  | Yes vs. NO |  | 1.186 (0.357; 3.941) |  | 0.7807 |  | <0.001 (<0.001; >999.999) |  | 0.9781 |
| Pre-term baby (<37 weeks) in previous pregnancies |  | Yes vs. NO |  | 4.704 (2.761; 8.014) |  | <.0001 |  | 1.661 (0.784; 3.519) |  | 0.1850 |

Footnote: OR, odds ratio; 95% CI, 95% confidence interval; Y, years.
