# Supplementary material for: Safety of reduced antigen content diphtheria-tetanus-acellular pertussis vaccine when administered during pregnancy as part of the maternal immunization program in Brazil: a single center, observational, retrospective, cohort study
Source: Hum Vaccin Immunother. 2019 Jun 20;15(12):2873–81. doi: 10.1080/21645515.2019.1627161 (PMC6930109; doi:10.1080/21645515.2019.1627161)
Supplement: Supplemental Material [file khvi-15-12-1627161-s001.zip › Supplementary table 3.docx]

**Supplementary table 3.** Congenital anomalies reported in neonates born to women from the exposed and unexposed cohorts

|  |  | **Exposed cohort**  **N=1199** | |  | **Unexposed cohort**  **N=1259** |
| --- | --- | --- | --- | --- | --- |
| **Congenital anomalies** |  | Numbers of events | Number of vaccine-related events |  | Numbers of events |
| Polydactyly |  | 1^a^ | 0 |  | 5^b^ |
| Sacral dimple |  | 0 | 0 |  | 5^c^ |
| Fetal multicystic kidney |  | 0 | 0 |  | 1 |
| Trisomy 21 |  | 0 | 0 |  | 2 |
| Hydrocephalus |  | 0 | 0 |  | 1 |
| Kidney malformation |  | 0 | 0 |  | 1 |
| Gastroschisis |  | 0 | 0 |  | 2^d^ |
| Rubella |  | 0 | 0 |  | 1 |
| Hypospadias |  | 1 | 0 |  | 1 |
| Anencephaly |  | 0 | 0 |  | 2^e^ |
| Microcephaly |  | 2 | 0 |  | 0 |
| Oculo-auriculo-vertebral spectrum |  | 1 | 0 |  | 0 |
| Bilateral preauricular sinus |  | 0 | 0 |  | 1 |
| Congenital Lung malformations |  | 0 | 0 |  | 1 |
| Cardiopathy |  | 0 | 0 |  | 1 |
| Congenital clubfoot |  | 1 | 0 |  | 1 |

Footnote: N, total number of participants (some cases presented with more than one event). Since events were recorded verbatim in the database, wording differs. ^a^ recorded as polydactyly of lower limb; ^b^ of which 1 recorded as left hand polydactyly; 1 as polydactyly of fingers congenital; 1 as appendix supernumerary-lower members right and left; and 1 as supernumerary on hands; ^c^ of which 1 recorded as sacral dimple congenital; ^d^ of which 1 recorded as fetal gastroschisis; ^e^ of which 1 recorded as Anencephalia (Portuguese for Anencephaly)
