# Supplementary material for: Safety of reduced antigen content diphtheria-tetanus-acellular pertussis vaccine when administered during pregnancy as part of the maternal immunization program in Brazil: a single center, observational, retrospective, cohort study
Source: Hum Vaccin Immunother. 2019 Jun 20;15(12):2873–81. doi: 10.1080/21645515.2019.1627161 (PMC6930109; doi:10.1080/21645515.2019.1627161)
Supplement: Supplemental Material [file khvi-15-12-1627161-s001.zip › Supplementary table 4.docx]

**Supplementary table 4**. Cumulative incidence of pregnancy-related adverse events and neonatal adverse events of interest by study year in current pregnancy in the unexposed cohort (total enrolled cohort)

| **Adverse event/ Birth outcome** | **Study year** | **n/N** | **Incidence proportion per 1000**  **(95% CI)** |
| --- | --- | --- | --- |
| Gestational diabetes | Sep2012-Aug2013 | 10/615 | 16.26 (7.80; 29.90) |
|  | Sep2013-Aug 2014 | 12/633 | 18.96 (9.80; 33.11) |
| Pregnancy-related hypertension | Sep2012-Aug2013 | 14/615 | 22.76 (12.45; 38.19) |
|  | Sep2013-Aug 2014 | 17/633 | 26.86 (15.64; 43.00) |
| Pre-Eclampsia | Sep2012-Aug2013 | 13/615 | 21.14 (11.26; 36.15) |
|  | Sep2013-Aug 2014 | 17/633 | 26.86 (15.64; 43.00) |
| Eclampsia | Sep2012-Aug2013 | 0/615 | 0.00 (0.00; 6.00) |
|  | Sep2013-Aug 2014 | 0/633 | 0.00 (0.00; 5.83) |
| HELLP Syndrome | Sep2012-Aug2013 | 1/615 | 1.63 (0.04; 9.06) |
|  | Sep2013-Aug 2014 | 0/633 | 0.00 (0.00; 5.83) |
| Vaginal hemorrhage | Sep2012-Aug2013 | 16/615 | 26.02 (14.87; 42.25) |
|  | Sep2013-Aug 2014 | 3/633 | 4.74 (0.98; 13.85) |
| Preterm birth | Sep2012-Aug2013 | 66/615 | 107.32 (83.00; 136.53) |
|  | Sep2013-Aug 2014 | 55/633 | 86.89 (65.46; 113.10) |
| Small for gestational age | Sep2012-Aug2013 | 31/615 | 50.41 (34.25; 71.55) |
|  | Sep2013-Aug 2014 | 31/633 | 48.97 (33.27; 69.51) |

Footnote: CI, confidence interval; N, number of participants with delivery date in a given category of unexposed cohort; n, number of participants where at least one event occurred (1) between index date and date of delivery for pregnancy related adverse events and (2) after index date for birth outcome events (preterm birth and small for gestational age). Vaginal hemorrhage, includes ante-partum, intra-partum and post-partum hemorrhage.
